# Trends in sugar supply and consumption in Australia: is there an Australian Paradox?

**DOI:** 10.1186/1471-2458-13-668

**Published:** 2013-07-18

**Authors:** Wavne Rikkers, David Lawrence, Katherine Hafekost, Francis Mitrou, Stephen R Zubrick

**Affiliations:** 1 Telethon Institute for Child Health Research, Centre for Child Health Research, The University of Western Australia, PO Box 855, West Perth, WA, 6872, Australia

**Keywords:** Public health, Sugar, Obesity, Food supply

## Abstract

**Background:**

High consumption of refined carbohydrate, in particular sugar, has been identified as a possible contributory factor in greater risk of excess weight gain. In spite of data limitations, one recent paper suggests that Australian sugar consumption has decreased over the same time period that obesity has increased, a so called ‘Australian Paradox’. Given the significant public health focus on nutrition, we aimed to estimate Australian sugar supply and consumption over recent decades, to determine whether these data could be used to make any conclusions about sugar’s role in obesity.

**Methods:**

Foods high in sugar were identified. Data relating to sugar supply and consumption from 1988 to 2010 were obtained from multiple sources. Using these data we attempted to generate a time series estimate of sugar in Australia’s food supply.

**Results:**

Australia produces and exports sugar from sugar cane and the sugar in imported foods has received little attention*.* We were unable to produce a reliable and robust estimate of total sugars in the Australian diet due to data limitations and a lack of current data sources. However, available Import data showed large increases in the volume and value of imported sweetened products between 1988 and 2010 to over 30 grams of sugar per person per day. Value estimates of local production of sweetened products also show substantial increases in this period.

**Conclusion:**

The Australian Paradox assertion is based on incomplete data, as it excludes sugar contained in imported processed foods, which have increased markedly. A major Australian public health target is to improve the quality of the food supply, and actions have been set in terms of achieving broader environmental changes. However, evaluation of progress is hampered by lack of high quality data relating to supply and consumption. We recommend the regular collection of comprehensive food supply statistics, which include both local production and imports. This would provide an inexpensive addition to survey data and could assist in monitoring sugar consumption trends in food supply. Such information would also help inform public health policy.

## Background

“The physical and emotional health of an entire generation and the economic health and security of our nation is at stake.” In announcing the Let’s Move initiative in the US in 2010, Michelle Obama used these words to describe the impact that obesity is having on Americans. These words might apply equally in the Australian context.

The prevalence of obesity in Australia is increasing at a faster rate than in other OECD countries, even the USA [1]. In the National Preventative Health Strategy the Australian Government lists obesity, along with tobacco and harmful use of alcohol, as one of the top three preventable health challenges facing the nation. The Australian government’s key strategy to address this issue is to “support innovation that helps build the evidence base” from which to guide the development of large scale interventions [2].

According to the Australian Bureau of Statistics (ABS) in 2011–12 nearly 42% of men and 28% of women were assessed as being overweight, while 28% of men and women respectively were assessed as being obese [3]. Not only have our rates of obesity increased more quickly than in any other OECD country but the proportion of overweight and obese adults in Australia is predicted to continue rising over the next 10 years [4]. As in other Western countries, this trend represents a threat to both the health and the economy of Australia. If these predictions related to obesity are accurate, it is likely that Type 2 diabetes will grow to become the leading cause of disease burden for males and the second leading cause for females by 2023 [2]. An additional concern is the increasing proportion of children who are classified as overweight or obese. Childhood overweight and obesity can increase risk of long term conditions such as Type 2 diabetes, heart disease, hypertension and stroke [5]. Most current treatments for overweight and obesity are ineffective and prevention of excess weight gain is preferable [6].

Whilst the primary drivers of excess weight gain and the relative importance of various dietary factors in obesity remain contentious, high consumption of refined carbohydrate, particularly sugar, has been identified as a possible contributory factor. Much research has focused on the relationship between high consumption of sugar and the risk of weight gain and poor health outcomes [7]. This body of evidence details the potential links between chronic and high consumption of sugars and increased risk of poor health.

In addition to plausible biological links between sugar consumption and excess weight gain, research from the USA provides temporal links between increased consumption of sugars and the increasing prevalence of obesity [8,9]. However, recently Barclay and Brand-Miller have suggested that Australian consumption of refined sugars has decreased over the last 30 years while rates of overweight and obesity have increased [10]. The authors labelled this relationship an *Australian Paradox* and suggested, based on these findings, further public health efforts to reduce sugar intake are unlikely to result in reductions in the prevalence of obesity in Australia.

Barclay and Brand-Miller obtained annual apparent consumption data from the Food and Agriculture Organisation (FAO) to create a time series estimate of sugar consumption from 1980 to 2003. This estimate suggests that there has been a substantial decline in sugar consumption in Australia over this time period. Traditionally, sugar supply estimates have been calculated based on the size of the domestic sugar crop minus sugar exports, as Australia has imported very little raw sugar until five years ago, when the value of imports rose dramatically. In one year alone, 2008 to 2009, the value of imports rose by nearly 280% to over $33 m. However, imports of processed foods into Australia have increased and we were unsure whether Barclay and Brand-Miller included this in their estimates.

Barclay and Brand-Miller presented additional data from nutrition surveys and industry sales data that suggest increasing sugar consumption from the mid-80s through the 1990s, although this may have peaked by 2005 and there may have been a slight decline in sugar consumption since then. This is consistent with recently published industry data suggesting a recent small decline in per capita sugar consumption [11]. However the prevalence of overweight and obesity in adults and children increased markedly in Australia between 1985 and 1995 and it is the ecological correlation between sugar consumption and prevalence of overweight and obesity that is at the core of the question of the existence of an Australian paradox.

Therefore, this paper aimed to: 1) verify whether reliable estimates of sugar supply and consumption within Australia over the last 20–30 years could be calculated with available data; 2) determine whether these data could be used to draw accurate and consistent conclusions about sugar’s effect on obesity; and, 3) conclude whether there is sufficiently robust data to support the existence of an Australian Paradox.

## Methods

### Data search strategy

We first identified food and beverages high in sugar (sucrose) content. We obtained lists of foods and beverages containing sugar using the NUTTAB 2010 Online Searchable Database on the Food Standards Australia New Zealand website [12]. These lists were confined to those food products imported into Australia which have relatively high sugar content (≥ 15 g sucrose per 100 g), such as cordials or confectionery, and those with medium sugar content (5 < 15 g sucrose per 100 g), such as soft drinks, which are likely to be imported and consumed in large quantities. Foods were then grouped into categories reflecting manufacturing and trade classifications [13]. These included: raw and refined sugar; jams and spreads; soft drinks, syrups and cordials; fruit drinks and fruit juices; fruit and vegetable processing; baked products such as bread, cakes, biscuits and pastries; chocolates and confectionery; and ice-cream. Alcoholic beverages were not included in the scope of this research. The main time period for which information was sought covered 1988 to 2010.

High fructose corn syrup was not identified as a separate category for data acquisition or analysis, as its use as a nutritive sweetener in Australia is limited.

### Data sources and acquisition

FAO Food Balance Sheets and [14] the F.O. Licht’s World Sugar Yearbook 2011 [15] were used to obtain estimates of per capita sugar consumption. Australian Food Statistics publications from 2000 to 2011 were used to source import figures for a range of food and beverage products containing added sugar [16]. The State of the Industry Report 2010 was used to source information on the sugar industry, food and beverage manufacturing data and import data [17].

Unpublished trade statistics on the importation and exportation of a variety of foods into Australia from 1988 to 2010 were obtained from the ABS (ABS, Customised reports, 2011). Due to the fact that this key data source was not available prior to 1988, we decided to restrict the time period of our entire analysis to the period 1988 to 2010.

Detailed manufacturing data are no longer directly collected in Australia on an ongoing or consistent basis, therefore, we used estimates of production contained in the ABS Australian Supply Use tables from the Australian National Accounts Input–output Tables Electronic Publications 1994–2008 as the best available measures of local production of a range of foods [18]. Australian production data for the time period 1998 to 2008 were obtained. It should be noted that the Input–output Tables are not designed by the ABS to be used to measure accurately individual commodities such as food items nor are they meant to be used as a time series. Consequently the estimates at this level should be used with caution. In addition, IBIS World reports on fruit drink, fruit juice and soft drink manufacturing were also accessed in order to corroborate data from other sources [19].

We used a number of classifications related to the production of statistics on food products. These included the Harmonised Tariff Item Statistical Classification (HTISC) [13]; the Working Tariff system for international trade data [20]; the Australian New Zealand Industry Classifications (ANZSIC) for 1993 [21] and for 2006 [22]; and the Input–output classification system used in the Australian National Accounts [18]. Imports of raw and refined sugars, as well as processed foods and beverages with high sugar content were obtained for the period 1988 to 2010 using both ANZSIC and HTSIC classification systems. Time series produced using the ANZSIC classifications should be interpreted with knowledge that there is a break in series between the 1993 and 2006 versions of the classifications.

For comparative purposes we also obtained estimates of Australian sugar availability over the past three decades from the FAO food balance sheets [14].

### Data treatment

In order to check consistency of data across sources, comparisons of data from different sources were made between items of similar description. We paid particular attention to those sources using data compiled utilising different classification systems.

Import values were deflated using the Food and Beverage category of the Import Price Index (IPI) [23] to convert all values to constant 1988 dollars using the following formula.

$$X\left(t\right)=\rho \left(t\right)*\frac{\mathrm{IPI}\left(t\right)}{\mathrm{IPI}\left(b\right)}$$

where *X* = Constant price; *t* = current time period; *ρ* = current price; *b* = 1988

Deflated values were also expressed as dollars per capita to account for changes in the size of the Australian population over this time period.

We estimated the imported quantity of total sugar and sugar in processed products from 1988 to 2010 using both ANZSIC and HTISC classifications. Where quantity data were missing, we used the recorded price to estimate the quantity figure. With the use of Australian dietary survey data [24] we estimated the proportion of sugar contained in each classification category. We then calculated a best estimate of the quantity of sugar contained in imported processed products using both the ANZSIC and HTISC classifications. Using population data from the ABS [25], we calculated the per capita per year quantity for sugar contained within imported and exported processed food and beverages.

## Results

**Figure 1:**
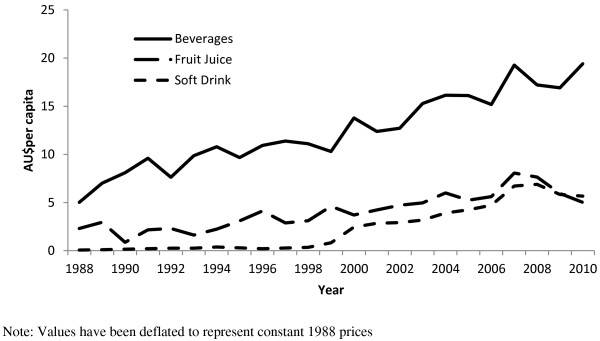
Estimated per capita sugar imports in processed food and beverages (grams per day).

If all forms of sugar in the diet are taken into account, such as refined sugar as well as sugar added to manufactured or processed food and drinks, the estimates of these sugar imports into Australia have shown a steady increase from 1988 to 2010. We used total production imports to calculate per capita per day consumption for total sugar imports and sugar imports from processed products high in sugar content (Figure 1). The possible concomitant increases in imports of products with lower sugar content over the same period would be expected to add to the total sugar in imported products.

**Figure 2:**
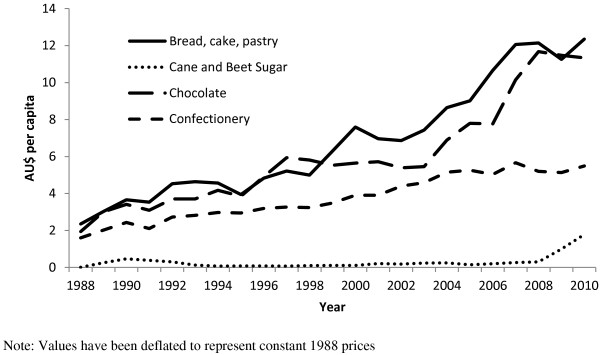
**Estimated per capita Major imports (AU$ per capita) of high sugar beverages 1988 – 2010 (HTISC classification).** Note: Values have been deflated to represent constant 1988 prices.

**Figure 3:**
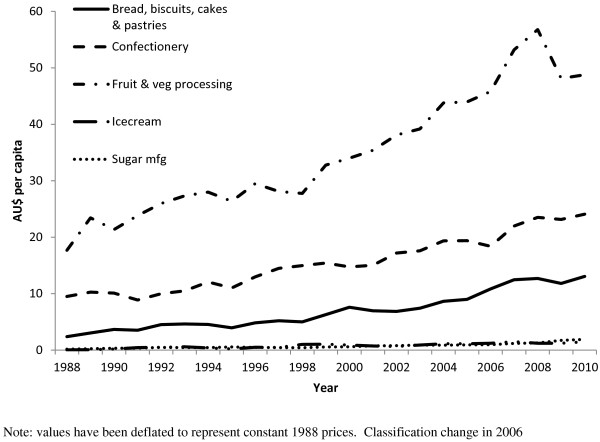
**Estimated per capita Major imports (AU$ per capita) of high sugar foods 1988 – 2010 (HTISC classification).** Note: Values have been deflated to represent constant 1988 prices.

When we looked at data compiled using the HTISC, we found that imports for the chosen range of sweetened food and beverage products showed substantial increases in volume and value over the period 1988 to 2010. For example, the value of imports of preparations for making non-alcoholic beverages (i.e. syrups used in the preparation of soft drinks) increased from $83 m in 1988 to $434 m in 2010. Other beverages such as fruit and vegetable juices rose from $38 m in 1988 to $112 m in 2010. Imports of sugar confectionery in 1988 were $26 m increasing to $123 m in value in 2010, and bread, pastry, cakes and biscuits increased at an even greater rate from $39 m in 1988 to $276 m in 2010. Chocolate imports paralleled this growth, rising from $32 m in 1988 to a value of $253 m in 2010. Per capita estimates for these commodities are included in Figure 2 and Figure 3.

**Figure 4:**
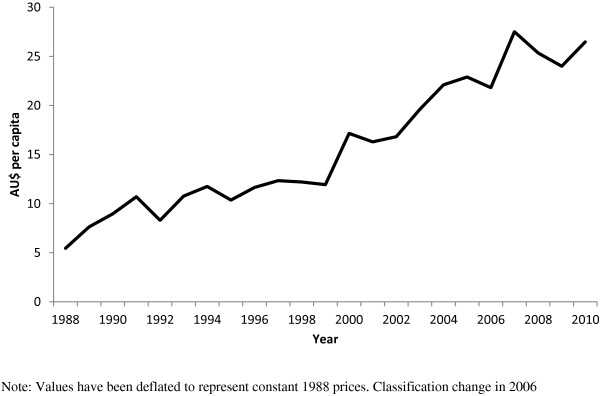
**Estimated per capita Major imports (AU$ per capita) of high sugar foods 1988 – 2010 (ANZSIC classification).** Note: values have been deflated to represent constant 1988 prices. Classification change in 2006.

**Figure 5:**
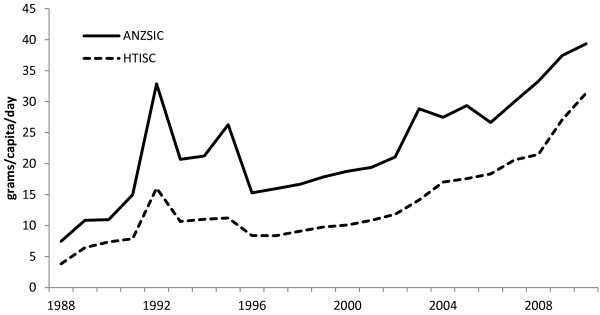
**Estimated per capita Soft drink imports (AU$ per capita) 1988 – 2010 (ANZSIC classification).** Note: Values have been deflated to represent constant 1988 prices. Classification change in 2006.

Examination of import data compiled using the ANZSIC classification system, as opposed to the HTISC, showed similar increases for comparable food (Figure 4) and beverage (Figure 5) products across the same time period.

**Figure 6:**
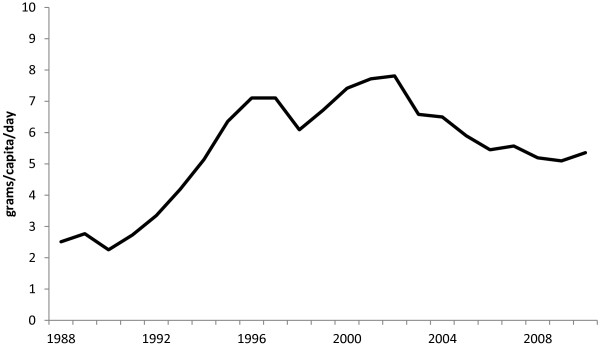
Estimated per capita sugar exports in processed food and beverages (grams per day).

While exports of highly processed food and beverage products have increased over the same time period, the increases have been minimal in comparison to imports of equivalent foods, In 2010 there were an estimated 6 g of sugar per capita per day in highly processed food products exported from Australia (Figure 6) compared with 30 g of sugar per capita per day imported into the country via similar products.

The value estimates of local production for a variety of foods and beverages containing added sugar also indicated an increasing trend (Table 1).

**Table 1:** Australian supply of various foodstuffs from 1998 to 2008 ($ million)

	**1998-1999**	**2001-2002**	**2004-2005**	**2005-2006**	**2006-2007**	**2007-2008**
Flour mill products and cereal foods	3276	3748	3979	3933	4234	5084
Bakery products	3373	5230	5528	5749	6130	6590
Confectionery	1453	2180	2240	2318	5093	6060
Soft drinks, cordials and syrups	2550	3196	3666	4061	4581	4811

Note: Values are reported in constant price.

## Discussion

The most commonly used measures of a country’s per capita sugar consumption are the estimates contained in the FAO Food Balance Sheets [14]. Even though analyses of various information sources show that Australians remain relatively high consumers of sugar when compared with other countries [26], the FAO estimates show that our sugar consumption appears to have plateaued in recent years. However, these estimates do not represent the total consumption of sugar by Australians and caution should be exercised in using them for this purpose. We contacted senior methodologists at the FAO head office and confirmed that when calculating per capita sugar consumption, the FAO does not include imported, highly processed foods or beverages which contain added sugar. According to the FAO handbook: ‘The definition of a complete list of potentially edible commodities presents virtually insurmountable difficulties - both conceptual and statistical… Generally, food balance sheets are constructed for primary crops, livestock and fish commodities up to the first stage of processing in the case of crops’ [27].

The FAO Food Balance Sheets focus on the production and trade of raw food products and therefore do not account for sugars contained in processed, imported food products. Trade statistics show that Australian food exports are mainly primary produce while imports comprise predominantly processed food and beverages. Methodology that relies on local production of, and trade in, primary produce only can give an increasingly inaccurate picture of the Australian food supply, as our importation of processed food products continues to grow. It is worth noting that Australia is one of the world’s biggest producers of sugar and more than 80% of our sugar crop is exported [28]. It is reasonable to assume that a proportion of this sugar is re-imported in the form of manufactured foods, such as nutritively-sweetened beverages, confectionery, ice cream and baked goods. In the fifty years to 1998–99, the amount of sugar consumed in manufactured foods more than doubled from 16.3 kg to 33.9 kg per capita, which indicated an increasing trend towards a preference for manufactured foods while the consumption of refined sugar for home use declined [29]. The value of imports into Australia of manufactured sweetened foods has grown substantially over the last 20 years, and we conclude this should be taken into account in the overall consumption of sugar. Even allowing for a general deflation factor of 17% in these values (as measured by the Food and Beverages item in the Import Price Index over this same time period [30]), it is clear from available trade data that the increases over the last 20 years in imports of foods containing added sugars are large and represent an increasing contribution to the dietary sugar available to the Australian population.

It is reasonable to conclude from this information that the Australian Paradox assertion is not supported by existing data, since it appears that per capita sugar consumption figures that show a decline in sugar consumption during the 1980s and 1990s do not include sugar contained in imported processed food products.

### Data limitations

In our attempts to determine whether available sources of data on sugar consumption, production and trade are reliable, we identified a number of data limitations which make drawing definitive conclusions about sugar problematic.

While our data suggest an apparent increase in consumption of sugar in Australia there were some limitations to our ability to make reliable estimates of supply or consumption over recent decades owing to poor data availability and quality. Clearly any researcher would suffer from the same limitations. Although it is common practice to source data widely for research, we found it troubling that different sources present very different estimates of apparently the same or similar items. This study identified a number of reasons for this seeming disparity. These included differences in the definitions used for the same item between either sources or researchers, the use of different classification systems from source to source to measure the same commodity, different time periods used in comparative literature when determining trends, the use of estimated versus collected data, the use of current versus constant prices when measuring changes over time, and differences in collection methodology employed by organisations which supply data for research purposes. Of particular concern was the absence of good quality data on volume and value of production of food items manufactured in Australia.

Although we found that the upward trend in imports was consistent across all sources, care should be taken when analysing data across any break in series due to possible compositional changes in items from series to series. For example, the ANZSIC 1993 version was updated in 2006 and any time series using this classification system may be affected. It is important to note also that trade estimates were limited by the inability to account fully for fluctuations in individual commodity prices. A further data limitation was caused by the mix of high and low sugar content items within certain classifications, meaning that some increases may have been attributed to the lower sugar content items.

**Figure 7:**
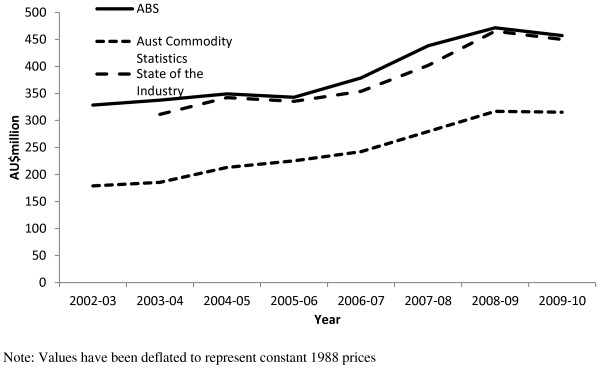
**Comparison of confectionery data (AU$’000) from various sources.** Note: Values have been deflated to represent constant 1988 prices.

The different definitions used in each of the classification systems create problems in combining data from different sources into an overall estimate. For example, a comparison of data reported for confectionery highlighted the lack of agreement across various data sources (Figure 7). Of the four sources we accessed, only the Australian Commodity Statistics and the Australian Food Statistics publications were found to contain the same figures. The differences between classifications can be attributed to different inclusions and exclusions for a particular category of food or beverage, even though the category titles may be very similar.

It should be noted that the estimates of local production are calculated by the ABS using slightly different methods and data inputs for each time period which limited our ability to make consistent comparisons over time. The break in series in 2006 due to an update in the ANZSIC classification may also have affected the figures. However, as an indicator series it provides compelling evidence that production of these foods and beverages is trending upwards.

It was not possible to locate reliable information on how much of the food consumed in Australia is manufactured locally or is imported. Nor was it possible for us to source information on how much of the sugar used in the manufacture of food in Australia is produced locally or is imported. Consequently, it was not possible to calculate with accuracy a time series estimate of the total amount of sugar available in the Australian diet from existing information. Therefore our estimates were based on the best available information. Lastly, available food supply data does not always account for all wastage of food; therefore estimates used for calculating consumption purposes may overstate the amount of food consumed.

However, even taking account of these limitations, available trade data suggests that the increases in imports of foods containing added sugars over the last two decades are large and represent a substantial and increasing contribution to the dietary sugar available to the Australian population.

### Need for new and improved data collections

Traditionally statistics on the sugar industry, and other agricultural production, have been collected in Australia for their economic importance. As the Australian economy has evolved and the size of Australia’s manufacturing sector has declined, many of these historical time series have been discontinued as the economic importance of these figures has diminished, along with the ability to collect reliable data on this sector. *Apparent Consumption of Foodstuffs* in Australia was last published in respect of the 1998/99 financial year.

In addition to their economic utility, these time series are now taking on additional public health significance. With the increasing rates of overweight and obesity, and associated chronic health conditions such as diabetes recognised as major population health issues, there is substantial attention focussed on changes in life styles and the food supply. Australia’s National Preventative Health Agency has set major targets for improving the quality of the food supply [2], and policy actions have been set to alter individual behaviours. Broader environmental changes are also likely and will reshape the food supply. Consequently, emphasis has also been placed on improving the relevant evidence base.

Apparent consumption figures could be an important adjunct to figures on individual consumption of foodstuffs. In Australia, individual consumption data have only been collected infrequently, because of the substantial costs involved. A significant concern with individual consumption data has been the trend for people to under-report what they eat; particularly when they know that what they are eating is unhealthy [31]. Compared with the collection of individual consumption data via national surveys, the compilation of apparent consumption data from agricultural production data and administrative records of imports is very cost effective. These data would provide a useful measure of national progress towards achieving preventative health goals, and would provide a means of confronting the shortcomings of individual consumption data via monitoring the overall supply. As the Australian government through ANPHA could be making significant public investment in programs to address obesity, including programs designed to alter the food supply, a modest investment in improving administrative data on the food supply is required to provide the necessary statistics to evaluate the impact of government interventions.

## Conclusion

Time series data on apparent consumption of sugars exist in Australia from the 1930s until 1999. For most of this period, the methodology of subtracting sugar exports from the size of the domestic sugar crop has produced a good estimate of the amount of sugar available in the domestic food supply. However, Customs data on imports of processed foods show that over the last 20 years there has been rapid and still increasing growth in imports of processed foods, many with high sugar content. The source of this sugar is unknown, but at least in some cases domestically grown sugar is exported for use by overseas food manufacturers and then returned to the country in the form of highly processed foods. Our conservative estimate suggests that at least one-sixth of the sugar in the domestic food supply is now imported into the country in this way. While production data alone suggest that from the growers’ perspective the amount of sugar sold into the domestic supply has been decreasing, this may not reflect a reduction in Australian consumption of sugar if this is offset by increases in imported refined foods containing sugar. This finding calls into question the existence of an Australian paradox as reported by Barclay and Brand-Miller. If our estimate of per capita daily sugars from imported foods were added to the FAO figures reported by Barclay and Brand-Miller, the result would be a substantially increasing trend in sugar available for consumption in Australia.

The authors of the Australian Paradox state that their analysis was based on three independent lines of evidence: national dietary surveys, apparent consumption data from the United Nations Food and Agricultural Organisation (FAO) and beverage industry data [10]. The data presented in their paper from Australian survey sources suggested constant or increasing consumption of sugar in the 1980s and 1990s, whereas, the authors appear to have relied on the FAO data to conclude that efforts by health authorities and government guidelines to limit sugar consumption in Australia are unwarranted and unlikely to impact on overweight, obesity and metabolic diseases. No ecological correlation can demonstrate a causal association between sugar consumption and obesity. However, the effect of imported foods containing sugar undermines claims of the existence of an Australian Paradox and show such a theory cannot be used as a basis for assuming that sugar consumption is not a significant factor in obesity in Australia.

This study identified that there remain substantive deficiencies in the statistics available on food supply and sugar consumption in this country. To support measuring progress in achieving national goals, we recommend re-establishing the regular collection and production of a comprehensive suite of national statistics on the Australian food supply. In light of the increasing contribution of imported foods in the food supply, we recommend that the methodology for the compilation of these figures be expanded to include both local production and imports. To this end, a revision of the classification used by Customs Australia for recording imports would be particularly valuable. The present classification, principally based on tariff structures, combines in single categories foods that would be considered of good as well as poor nutritional value, thus compromising the ability to monitor changes in the nutritional quality of the food supply.

## Appendix

## Response

By Alan Barclay and Jennie Brand-Miller

Email: awbarclay@optusnet.com.au and jennie.brandmiller@sydney.edu.au

Address: Australian Diabetes Council, 26 Arundel Street, Glebe, NSW 2037, Australia

School of Molecular Bioscience and Boden Institute of Obesity, Nutrition and

Exercise, University of Sydney, NSW 2006, Australia

In this issue of the *Journal*, Rikkers et al. attempt to estimate Australian refined sucrose supply and consumption over recent decades. They conclude that it is not possible to produce a reliable and robust estimate because of ‘data limitations and a lack of current data sources’.

The failure to recognise declining sugar concentration in Australian beverages may also apply to estimates of the sucrose content of imported foods. Indeed, it is not possible to determine precisely what proportion of imported soft drinks, chewing gums, chocolate and confectionery are manufactured with non-nutritive sweeteners and low digestibility sweeteners (e.g., polyols). We do know that ‘low sugar’ and ‘no added sugar’ products have become increasingly popular [8] and these are more likely to be imported than manufactured in Australia. The figure of 30 g sucrose/day from imported foods is therefore likely to be an overestimate.

**Figure 8:**
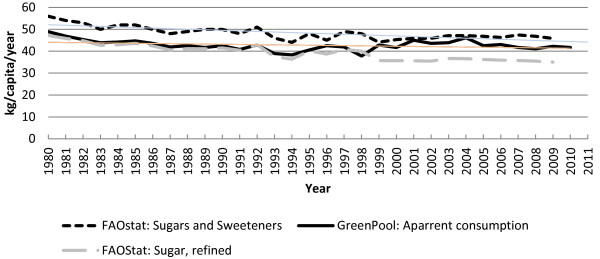
Comparison of estimated per capita apparent consumption of sugar for Australia from 1980-2011 [1,11].

The analysis by Rikkers et al. makes much of imported sources of sugar, but overlooks the export of sugar as both a value-added ingredient, and in certain categories of food that are high in added sugars (e.g., dairy). Historically, apparent consumption data from ABS has included both imports and exports in processed foods. FAOStat data for Australia are almost identical to ABS data until 1998–99 when reporting ceased (Figure 8), implying similar methodologies. The most recent FAOStat data for Australia show that sugar availability has continued to decline (Figure 8). The Green Pool analysis [1] extended the ABS apparent consumption of sugar data series from 1999 to 2011 (Figure 8). Their detailed analysis included 173 categories of imported products and 120 categories of exported products, while Rikkers et al. included fewer than 20 food categories and overlooked exports of value added ingredients. The Green Pool analysis concluded that apparent consumption of sugar declined from 1980–2011, i.e., a conclusion that is similar to the most recent FAOStat data.

Other limitations should be noted. In their analysis, Rikkers et al. were obliged to make assumptions about the cost of imported food items in order to derive an estimate of amount consumed. However, imported goods vary markedly in price depending on country of origin, but can be much more expensive than the local product (up to 10-fold more per litre in the case of soft drink). Underestimating the cost of imported products will overestimate the amounts consumed and therefore overestimate the sugar content from imported products. Similarly, food wastage is now higher than in the past with 34% of food now wasted at the consumer level [9]. National nutrition survey data, as cited in the *Australian Paradox*, provide the most precise data on food actually consumed.

Finally, Rikkers et al. should heed their own conclusion that we should not make too much of incomplete data. Thankfully, reliable data on the intake of added sugars by Australians will be generated by the 2011–12 National Nutrition Survey due for release later this year [10].

Nonetheless, their analysis suggests that *imported foods* are now a greater contributor to intake of refined sucrose than they were in the past. Common sense would suggest that’s true because over the past decade we have imported more foods in general, but this finding does not prove that added sugars intake from all sources is now higher than in the past. Indeed, new data indicate that Australia now exports more foods and ingredients containing refined sucrose than 10 years ago [1]. There is evidence that not only Australians, but Americans are consuming less refined sugars than a decade ago [2].

In 2011, Barclay and Brand-Miller [3;4] reported three separate lines of evidence indicating downward trends in added sugars intake over the same timeframe that the prevalence of overweight and obesity among Australians had dramatically increased. We referred to this inverse relationship as the *Australian Paradox*. Rikkers et al. claim that the *Australian Paradox* is based on incomplete data because the sources utilised did not incorporate estimates for imported processed foods. This assertion is incorrect. Indeed, national nutrition surveys, sugar consumption data from the United Nations Food and Agricultural Organisation (FAOStat), the Australian Bureau of Statistics (ABS) and Australian beverage industry data *all* incorporated data on imported products. The apparent consumption data presented in the *Australian Paradox* covered 1980–2003, the most recent data at the time*.* Although the data for the 4-year period 1999–2003 now appear to have been underestimated, they do not alter the underlying trend – per capita sugar consumption is still lower than it was in 1980, and during that time frame obesity rates trebled.

Rikkers et al. have also misinterpreted the results of national nutrition surveys in 1983 and 1995 by confusing total sugars with added sugars. These surveys indicate the percentage of energy from *total* sugars (a measure that includes the naturally-occurring sugars in fruit, vegetables and dairy foods) remained either the same or decreased from 1983 to 1995 (depending on the age group) [5,6]. However, the surveys demonstrated declines in “sugary products” that contribute refined added sugar against increasing intakes of fruit and vegetables, implying that the absolute intake of refined added sugars had declined over time.

Australian beverage industry data also suggest that the total amount of added sugar consumed in the form of soft drinks decreased from 1997–2006 [7]. Unfortunately, Rikkers et al. interpret the change in the volume of beverages as equivalent to change in sugar consumption, failing to recognise a decline in the *concentration* of added sugar in soft drinks. Manufacturers now sell soft drinks with as little as 3-5% sucrose vs 10-12% in the past. This critical information is not encapsulated by volume sales data, but by data on amounts of sugar used by the beverage industry (Figure six in the *Australian Paradox*).

## References (response)

[1] Green Pool Commodity Specialists. **Sugar Consumption in Australia**. Brisbane, Australia https://greenpoolcommodities.com/files/8113/4932/3223/121004_Sugar_Consumption_in_Australia_-_A_Statistical_Update_-_Public_Release_Document.pdf. 2012.

[2] White JS. **Challenging the fructose hypothesis: new perspectives on fructose consumption and metabolism**. *Adv Nutr* 2013 Mar 1;**4**[2]:246–56.

[3] Barclay A.W, Brand-Miller J. **The Australian Paradox: A Substantial Decline in Sugars Intake over the Same Timeframe that Overweight and Obesity Have Increased**. *Nutrients***3**, 491–504. 20-4-2011. MDPI Publishing, Basel, Switzerland.

[4] Barclay A.W, Brand Miller JC. **The Australian Paradox Revisited**. *Nutrients*http://www.mdpi.com/2072-6643/3/4/491/s3. 30-3-2012.

[5] Cobiac L. **Sugars in the Australian diet: results from the 1995 National Nutrition Survey**. *Nutrition* &*Dietetics* 2003;**60**[3]:152–73.

[6] Department of Community Services and Health. **National Dietary Survey of Adults: 1983**. Canberra: *AGPS*, 1987.

[7] Levy, G.; Tapsell, L. **Shifts in purchasing patterns of non-alcoholic, water-based beverages in Australia**, 1997–2006. *Nutr. Diet.* 2007, ***64***, 268–279.

[8] Food Standards Australia New Zealand. **Consumption of intense sweeteners in Australia and New Zealand: benchmark survey 2003**. Evaluation report series no. 8, *FSANZ*, 2004.

[9] United States Department of Agriculture, Economic Research Service **Sugar and Sweeteners Yearbook Tables.** 2012.

[10] Australian Bureau of Statistics. **Australian Health Survey**. Canberra, *ABS*. http://www.abs.gov.au/websitedbs/D3310114.nsf/home/AHS+Output+Strategy:+Work+program+and+timetable. Accessed 2/6/2013

[11] Food and Agricultural Organisation of the United Nations: **FAOSTAT**, **World Food Balance Sheet**. 2013. Accessed 1/6/2013

## Competing interests

The authors declare that they have no competing interests.

## Authors’ contributions

DL and WR conceived the design of the study. WR completed the analysis and wrote the initial draft. All authors contributed to the writing and approved the final manuscript.

## Pre-publication history

The pre-publication history for this paper can be accessed here:

http://www.biomedcentral.com/1471-2458/13/668/prepub
