# Correction: Trends in sugar supply and consumption in Australia: is there an Australian Paradox?

**DOI:** 10.1186/1471-2458-13-1134

**Published:** 2013-12-05

**Authors:** Wavne Rikkers, David Lawrence, Katherine Hafekost, Francis Mitrou, Stephen R Zubrick

**Affiliations:** 1 Telethon Institute for Child Health Research, Centre for Child Health Research, The University of Western Australia, PO Box 855, West Perth WA 6872, Australia

## 

This correction article relates to the response by Alan Barclay and Jennie Brand-Miller that was published as part of a research article [1] authored by Rikkers et al. During the publication process, errors were introduced into the formatting which affected the response only and caused the paragraphs to appear in the incorrect order. The research article reported by Rikkers et al. was not affected. A corrected version of the response has subsequently been published as a correspondence article [2]. BioMed Central apologize for any inconvenience caused.

## Pre-publication history

The pre-publication history for this paper can be accessed here:

http://www.biomedcentral.com/1471-2458/13/1134/prepub
